# Supplementary material for: Alternative carbon price trajectories can avoid excessive carbon removal
Source: Nat Commun. 2021 Apr 15;12:2264. doi: 10.1038/s41467-021-22211-2 (PMC8050196; doi:10.1038/s41467-021-22211-2)
Supplement: Supplementary file 1 — Supplementary Information [file 41467_2021_22211_MOESM1_ESM.pdf]

Supplementary Information

Alternative carbon price trajectories can avoid excessive carbon removal

Jessica Strefler*[1], Elmar Kriegler[1,2], Nico Bauer[1], Gunnar Luderer[1,3], Robert C. Pietzcker[1], Anastasis Giannousakis[1], Ottmar Edenhofer[1,3, 4]

[1]*Potsdam Institute for Climate Impact Research (PIK), Member of the Leibniz Association, PO Box 601203, 14412 Potsdam, Germany*

[2]*Universität Potsdam, August-Bebel-Straße 89, 14482 Potsdam, Germany*

[3]*Technische Universität Berlin, Straße des 17. Juni 135, 10623 Berlin, Germany*

[4]*Mercator Research Institute on Global Commons and Climate Change, Torgauer Straße 12–15, 10829 Berlin, Germany*

* Corresponding author, eMail strefler@pik-potsdam.de, phone +49 331 288 2475

Contents:

1. Supplementary model description
   1.1. Techno-economic data of reforestation and afforestation
   1.2. Techno-economic data of BECCS technologies
   1.3. Techno-economic data of DACCS
   1.4. Trade-offs between CDR technologies
2. Supplementary figures
3. Supplementary tables
4. Supplementary references

1. Supplementary model description

1.1. Techno-economic data of reforestation and afforestation

CO_2 emissions from deforestation and afforestation are calculated using marginal abatement cost curves. These cost curves are derived from the land-use optimization model MAgPIE (Model of Agricultural Production and its Impact on the Environment)[1,2] where land-based mitigation is incentivized by a price on GHG emissions from the land system. The objective function of MAgPIE is the fulfilment of food, livestock and material demand at minimum costs under socio-economic and biophysical constraints. While the GHG price renders deforestation and the conversion of pasture to cropland more costly, CO_2 removal through afforestation is rewarded and lowers the costs in the objective function of the MAgPIE model. Afforestation is implemented as managed regrowth of natural vegetation, thereby affecting vegetation, litter and soil carbon stocks. Carbon accumulation in living biomass follows sigmoidal tree growth curves based on a Chapman–Richards volume growth model, which is parameterized using vegetation carbon density of potential natural vegetation from the LPJmL model[3] and climate region specific mean annual increment (MAI) and MAI culmination age[4]. Soil and litter carbon densities are assumed to increase linearly over 20 years, starting from the weighted average carbon density of cropland and pasture[5,6].

1.2. Techno-economic data of BECCS technologies

Supplementary Table 1: techno-economic parameters of BECCS technologies

Technology (all with CCS)	Investment costs [US$2015/kW]	O&M costs [US$2015/GJ]	Conversion efficiency	Lifetime [years]	CO_2 Capture rate
**Biomass integrated gasification combined cycle power plant**	3150	5.59	0.28	40	80 %
**Biomass to hydrogen**	2040	6.80	0.55	35	90%
**Biodiesel production with Fischer-Tropsch**	3600	4.90	0.41	35	48%

1.3. Techno-economic data of DACCS

Direct air capture plants filter CO_2 directly from the ambient air. A variety of possible systems have been proposed, usually containing three main components: contactor, CO_2 absorbing material, regeneration system. The contactor must expose the CO_2 absorbing material to the ambient air and exchange saturated material for regenerated material. The CO_2 absorbing material must be able to pull large amounts of CO_2 from the atmosphere and release the CO_2 in the regenerating system. The released CO_2 is then captured and stored in geological storage formations. Depending on the absorbing material, temperatures of at least 800°C are needed for regeneration[7]. In the model, natural gas or H_2 can be used to generate the required heat. If natural gas is used, the resulting CO_2 emissions are assumed to be captured with a capture rate of 90%. Since the first demonstration plant has only started operating recently, data on costs and energy requirements is still uncertain. We rely on a literature review[7] for techno-economic parameterization, as specified in the table below.

Supplementary Table 2: techno-economic parameters of DAC

**Investment costs [US$2015/(tCO2/yr)]**	100
**O&M costs [US$2015/(tCO2/yr)]**	2.5
**Lifetime [years]**	20
**Electricity demand [GJ/tCO2]**	2
**Heat demand [GJ/tCO2]**	10

1.4. Trade-offs between CDR technologies

BECCS and afforestation compete for land which limits the total carbon dioxide removal potential for these two options. The marginal abatement cost curves for CO_2 emissions from deforestation and afforestation as well as the bioenergy supply curves were both derived from the MAgPIE model. The respective other technology was included in the necessary model runs, such that the land competition is taken into account.

BECCS, DACCS, and fossil CCS compete for geological storage. In REMIND, regional annual CCS is limited to 0.5% of the total available geological storage in that region. This limits total global CCS use to about 20 Gt CO_2/yr.

2. Supplementary figures

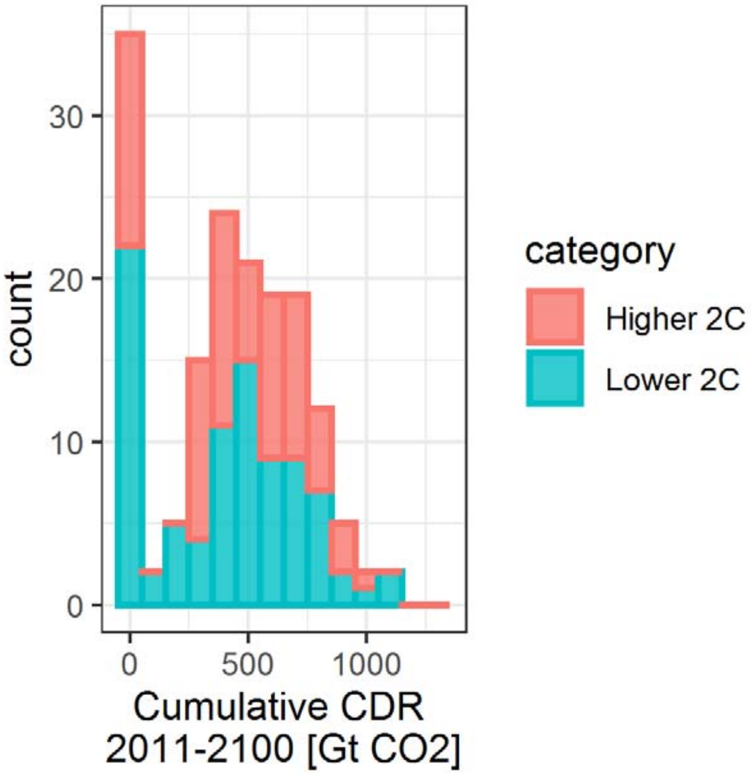

Supplementary Figure 1: Histogram of cumulative CDR use from 2011-2100 in 2°C scenarios in the IPCC Special Report on Global Warming of 1.5°C (SR1.5). We distinguish two different categories of 2°C scenarios: higher 2°C (probability between 50% and 66% of staying below 2°C, red) and lower 2°C (probability of at least 66% of staying below 2°C, blue), as defined in the database of the SR1.5 (https://data.ene.iiasa.ac.at/iamc-1.5c-explorer/#/login).

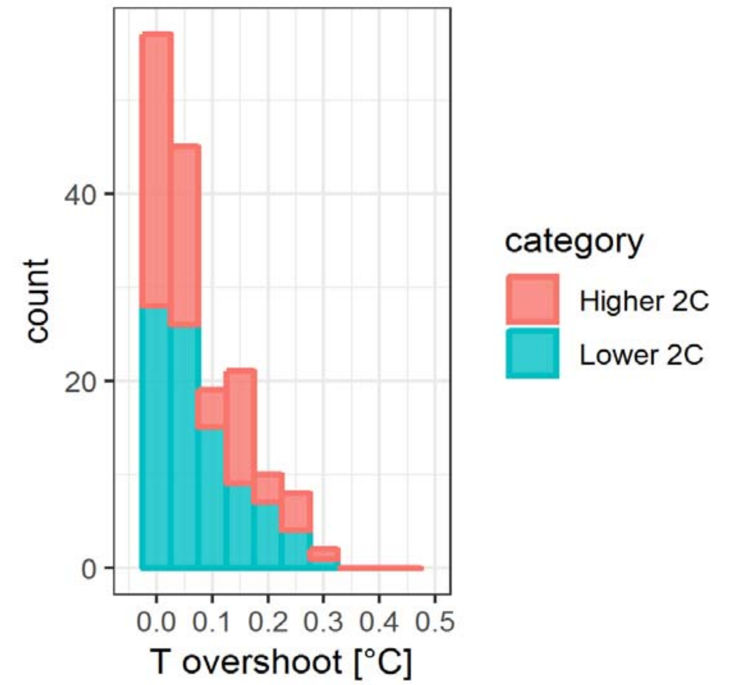

Supplementary Figure 2: Histogram of difference between peak temperature and end-of-century temperature in 2°C scenarios in the SR1.5. We distinguish two different categories of 2°C scenarios: higher 2°C (probability between 50% and 66% of staying below 2°C, red) and lower 2°C (probability of at least 66% of staying below 2°C, blue), as defined in the database of the SR1.5 (https://data.ene.iiasa.ac.at/iamc-1.5c-explorer/#/login).

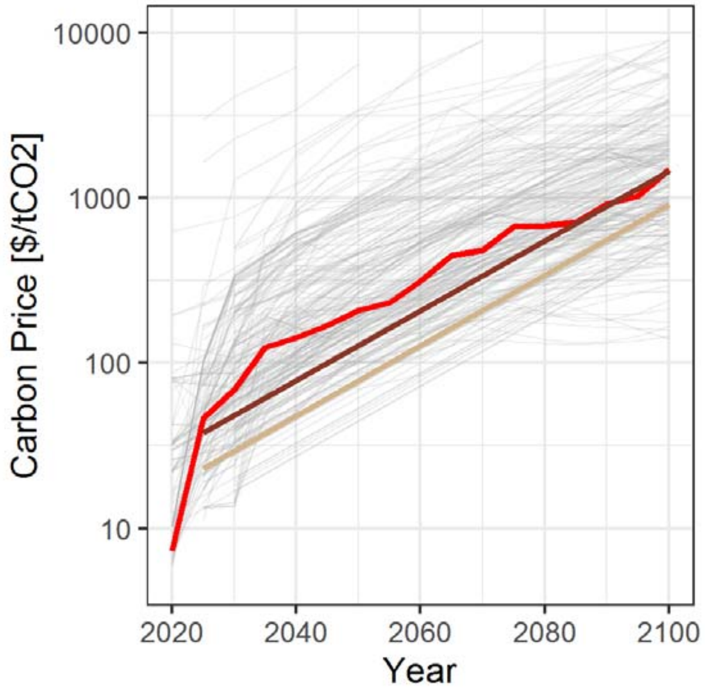

Supplementary Figure 3: Carbon price pathways in all scenarios in the SR1.5. The thick red line shows the median value of all SR1.5 scenarios (https://data.ene.iiasa.ac.at/iamc-1.5c-explorer/#/login). The dark and light brown lines show the Hotelling Below and Hotelling Overshoot scenarios, respectively, for comparison.

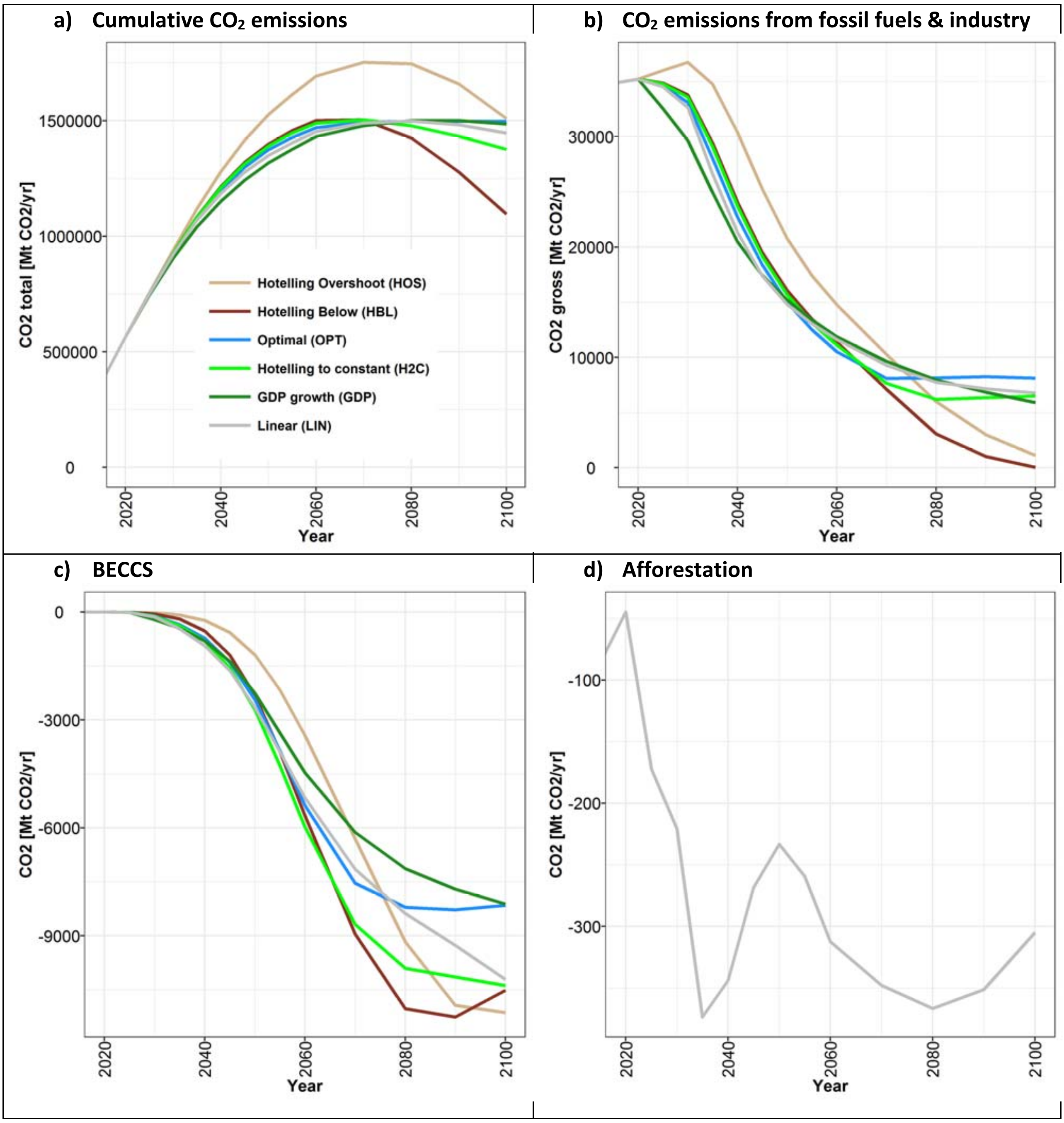

a) Cumulative CO₂ emissions
b) CO₂ emissions from fossil fuels & industry
c) BECCS
d) Afforestation
CO2 total [Mt CO2/yr]
CO2 gross [Mt CO2/yr]
CO2 [Mt CO2/yr]
Year
Hotelling Overshoot (HOS)
Hotelling Below (HBL)
Optimal (OPT)
Hotelling to constant (H2C)
GDP growth (GDP)
Linear (LIN)

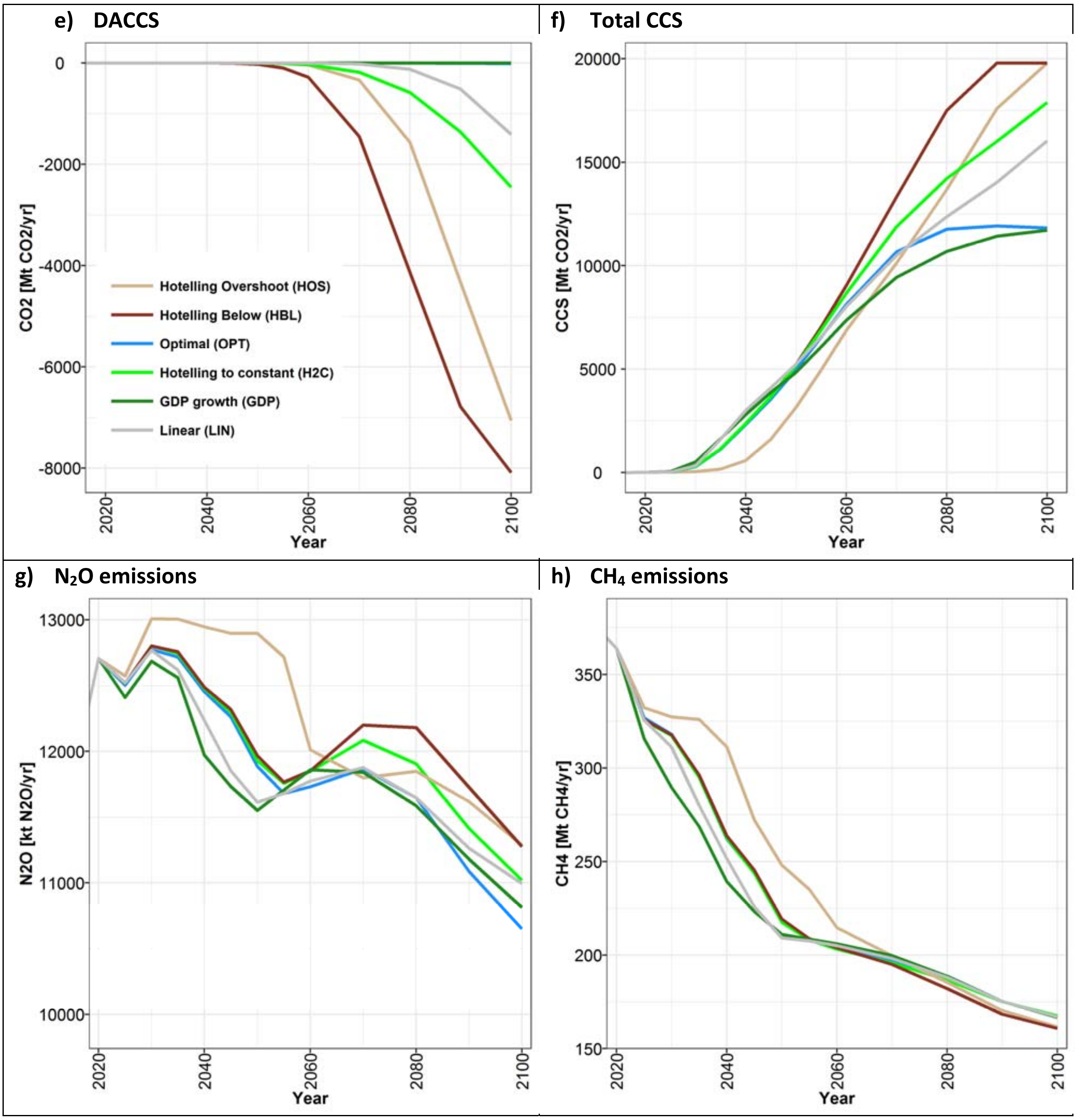

e) DACCS
f) Total CCS
CO2 [Mt CO2/yr]
0
-2000
-4000
-6000
-8000
Hotelling Overshoot (HOS)
Hotelling Below (HBL)
Optimal (OPT)
Hotelling to constant (H2C)
GDP growth (GDP)
Linear (LIN)
CCS [Mt CO2/yr]
20000
15000
10000
5000
0
2020
2040
2060
2080
2100
Year
g) N₂O emissions
h) CH₄ emissions
N2O [kt N2O/yr]
13000
12000
11000
10000
CH4 [Mt CH4/yr]
350
300
250
200
150

i) Cumulative discounted consumption loss until 2100 with respect to current policies

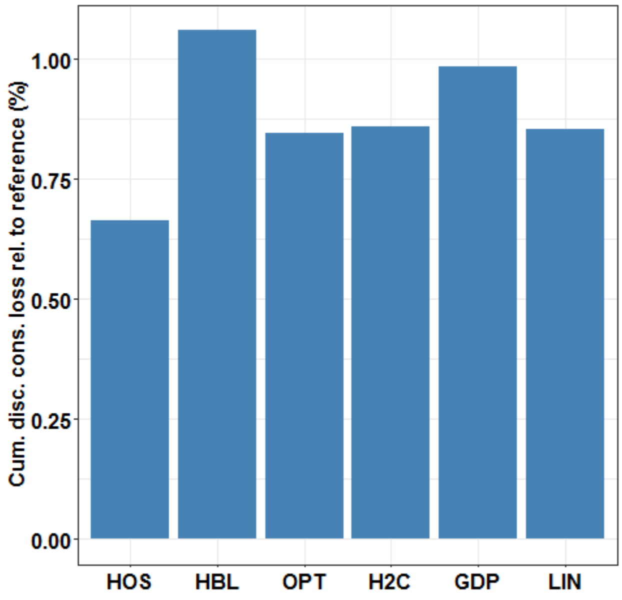

Supplementary Figure 4: Additional emission and CDR pathways and consumption losses for all six scenarios. a) cumulative CO_2 emissions, b) CO_2 emissions from fossil fuels and industry, carbon removed due to c) afforestation, d) BECCS, e) DACCS, f) total amount of CCS, g) global N_2O emissions, h) global CH_4 emissions, i) cumulative discounted (discount rate 5%, base year 2020) consumption loss as compared to a scenario with continued current policies until 2100 for the Hotelling Overshoot (light brown), Hotelling Below (dark brown), Optimal (blue), Hotelling to Constant (light green), GDP Growth (dark green), and Linear (gray) scenarios.

a) **Carbon price**

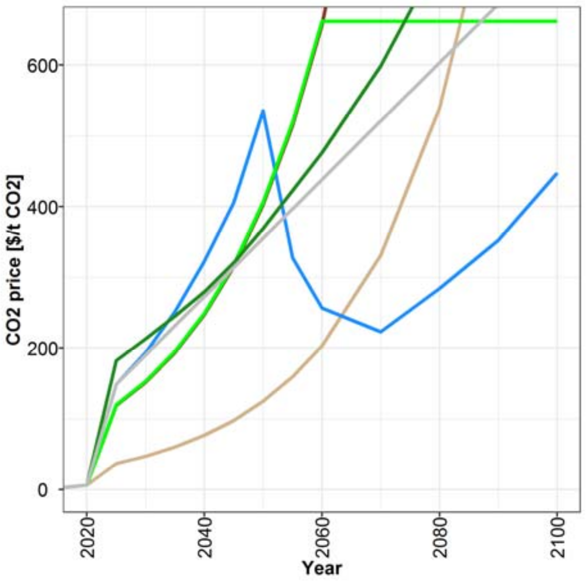

b) CDR deployment

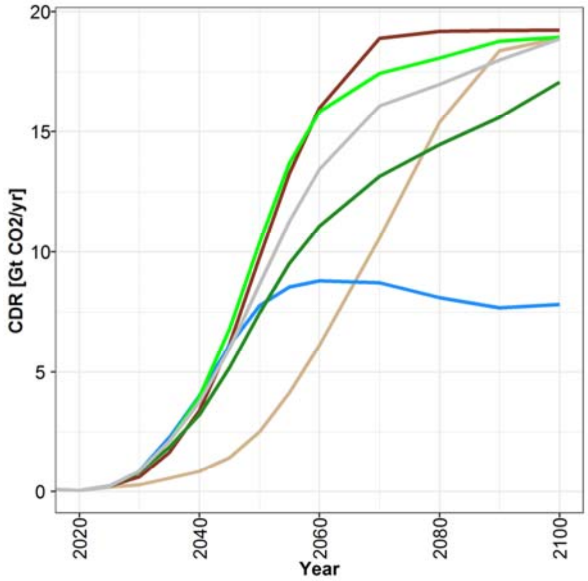

c) Total CO_2 emissions

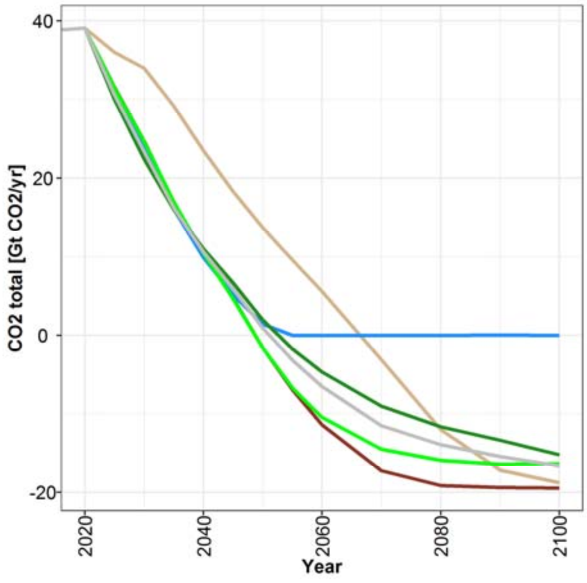

d) Global mean temperature increase

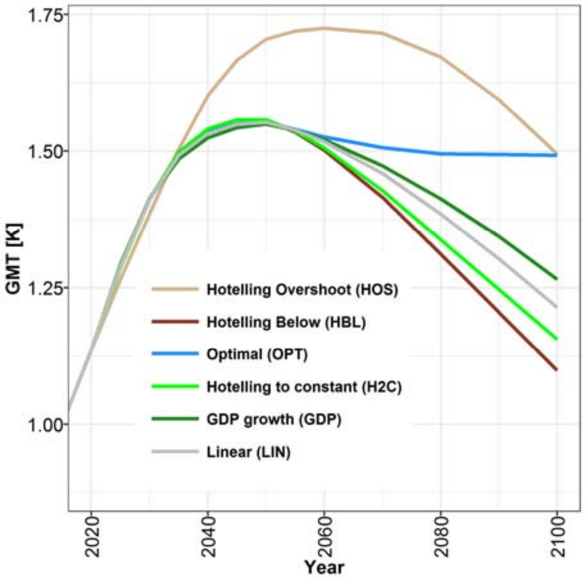

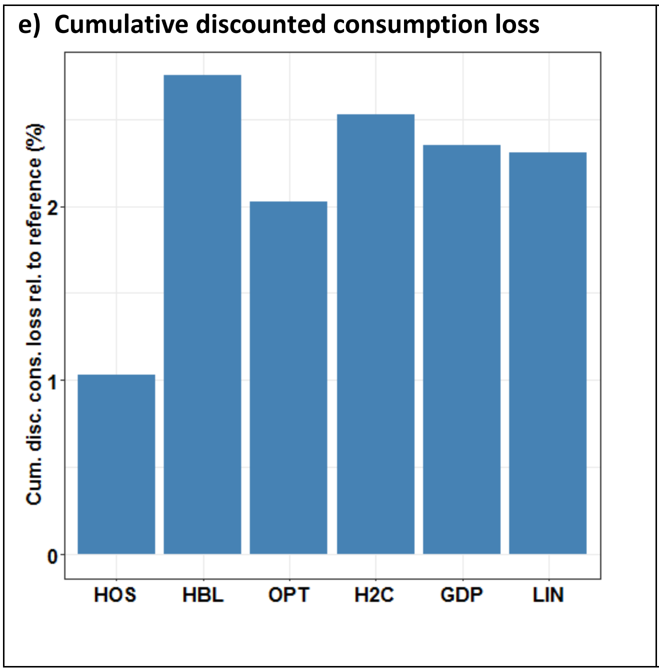

Supplementary Figure 5: Scenario characteristics as shown in Figure 1 for 1.5°C scenarios. Similar to Figure 1 we show a) Carbon price, b) CDR deployment, c) total CO_2 emissions, d) global mean temperature increase, and in addition e) cumulative discounted (discount rate 5%, base year 2020) consumption loss with respect to a scenario with continued current policies until 2100 for the scenarios Hotelling Overshoot (light brown), Hotelling Below (dark brown), Optimal (blue), Hotelling to Constant (light green), GDP Growth (dark green), and Linear (gray), but with a carbon budget of 670 Gt CO_2 from 2018 onwards, i.e. compatible with a 1.5°C target.

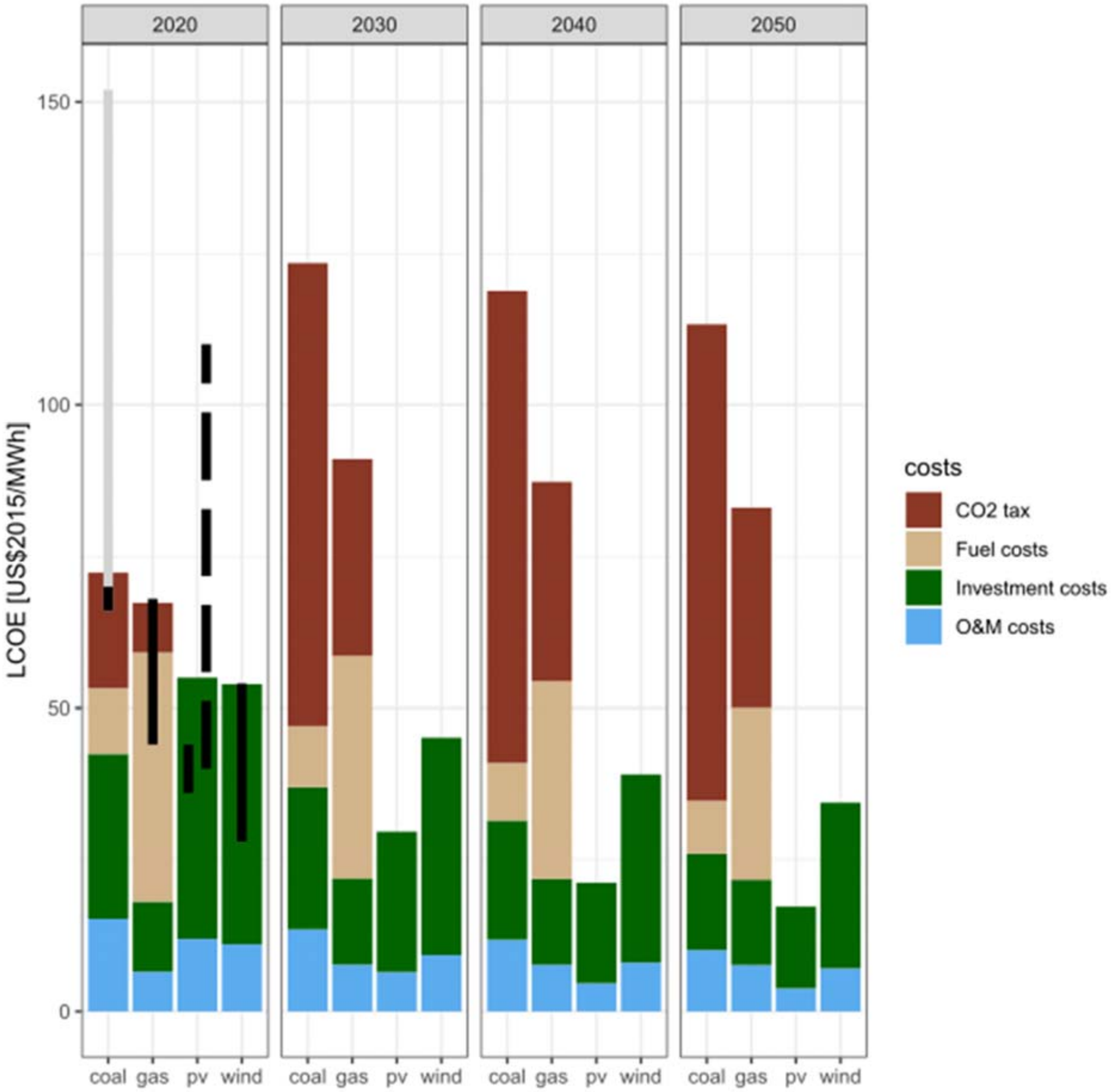

Supplementary Figure 6: Levelized costs of electricity production in the US from coal, natural gas, solar PV, and wind onshore in a REMIND scenario with continued current policies for 2020-2050. For comparison, the black lines gives the range of current prices based on Lazard[8] (solid) and Bolinger et al.[9] (dashed). The range of current prices from Lazard for coal includes plants with CCS; we would therefore concentrate on values on the lower end and marked the higher values in grey. LCOEs here consist of investment costs, operation and maintenance costs, fuel costs, and the costs for CO_2 emissions.

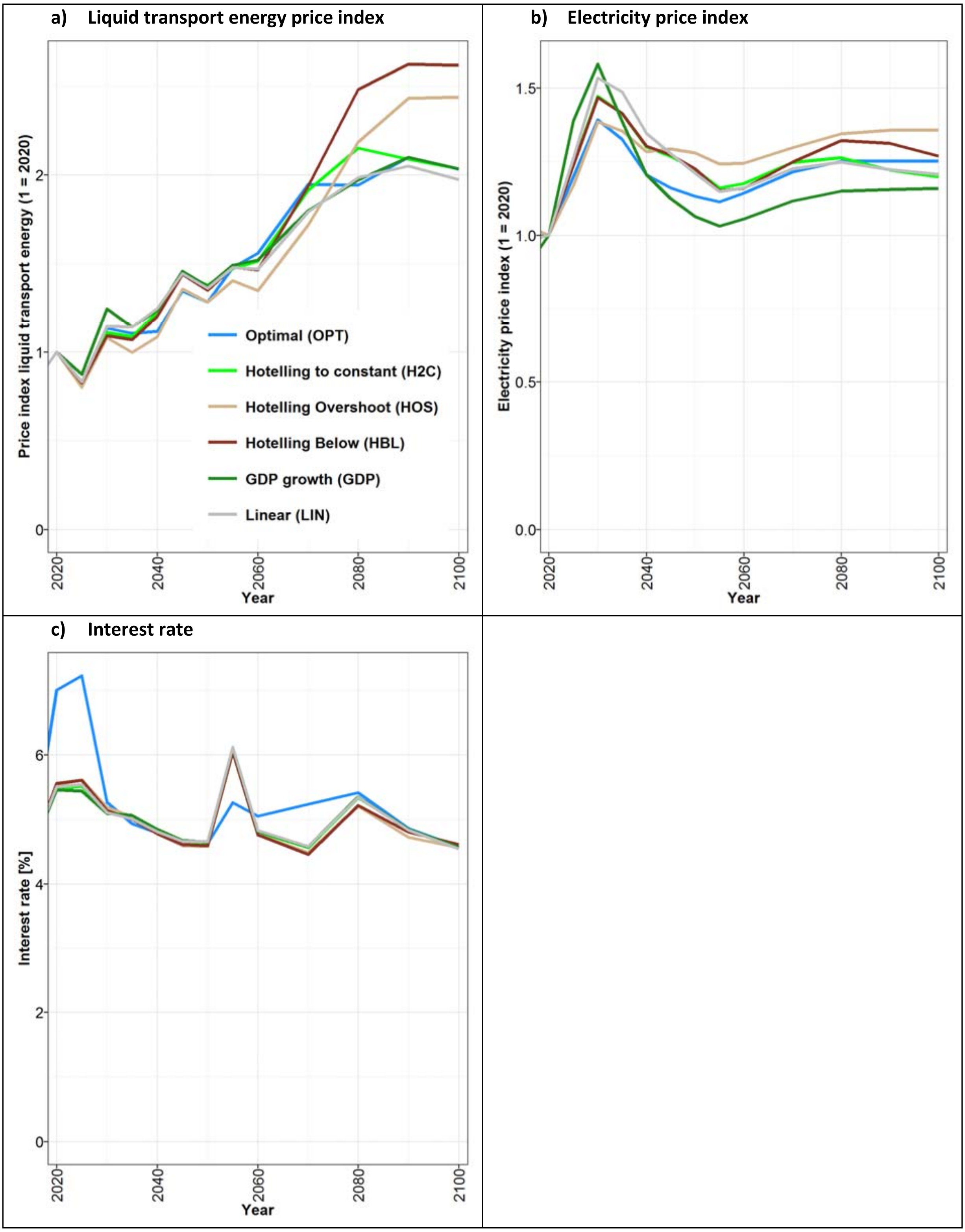

Supplementary Figure 7: Additional economic indicators. a) Liquid transport energy price index, b) electricity price index, c) interest rate for the scenarios Hotelling Overshoot (light brown), Hotelling Below (dark brown), Optimal (blue), Hotelling to Constant (light green), GDP Growth (dark green), and Linear (gray).

3. Supplementary Tables

Supplementary Table 3: Carbon price development for the three main scenarios.

**Carbon price [$/tCO$_2$]**	**2025**	**2030**	**2040**	**2050**	**2100**
**Hotelling Overshoot**	23	30	49	79	907
**Hotelling Below**	38	48	78	127	1460
**Optimal**	36	48	79	128	384

Supplementary Table 4: CDR deployment for the three main scenarios.

**CDR deployment [Gt CO$_2$/yr]**	**2025**	**2030**	**2040**	**2050**	**2100**
**Hotelling Overshoot**	0.18	0.24	0.58	1.4	18.5
**Hotelling Below**	0.18	0.28	0.87	2.6	18.9
**Optimal**	0.18	0.33	1.1	2.7	8.5

Supplementary Table 5: Total CO_2 emissions for the three main scenarios.

**Total CO_2 Emissions [Gt CO$_2$/yr]**	**2025**	**2030**	**2040**	**2050**	**2100**
**Hotelling Overshoot**	37.1	36.9	30.0	19.5	-17.4
**Hotelling Below**	35.9	33.9	23.4	13.7	-18.8
**Optimal**	35.7	33.2	22.1	12.8	0

4. Supplementary references

1. Popp, A. *et al.* Land-use protection for climate change mitigation. *Nat. Clim. Change* **4**, 1095–1098 (2014).
2. Stevanović, M. *et al.* Mitigation Strategies for Greenhouse Gas Emissions from Agriculture and Land-Use Change: Consequences for Food Prices. *Environ. Sci. Technol.* **51**, 365–374 (2017).
3. Schaphoff, S. *et al.* Contribution of permafrost soils to the global carbon budget. *Environ. Res. Lett.* **8**, 014026 (2013).
4. IPCC. *2006 IPCC Guidelines for National Greenhouse Gas Inventories*. (2006).
5. Humpenöder, F. *et al.* Investigating afforestation and bioenergy CCS as climate change mitigation strategies. *Environ. Res. Lett.* **9**, 064029 (2014).
6. Kreidenweis, U. *et al.* Afforestation to mitigate climate change: impacts on food prices under consideration of albedo effects. *Environ. Res. Lett.* **11**, 085001 (2016).
7. Broehm, M., Strefler, J. & Bauer, N. Techno-Economic Review of Direct Air Capture Systems for Large Scale Mitigation of Atmospheric CO2. *SSRN Electron. J.* (2015) doi:10.2139/ssrn.2665702.
8. Lazard. Lazard's Levelized cost of energy analysis-version 13.0. (2019). https://www.lazard.com/media/451086/lazards-levelized-cost-of-energy-version-130-vf.pdf

9. Bolinger, M., Seel, J. & Robson, D. *Utility-Scale Solar 2019. Empirical trends in project technology, cost, performance, and PPA pricing in the United States.* https://emp.lbl.gov/sites/default/files/lbnl_utility_scale_solar_2019_edition_final.pdf (2019).
